# *Streptococcus salivarius* Role as a Probiotic in Children’s Health and Disease Prophylaxis—A Systematic Review

**DOI:** 10.3390/life14121613

**Published:** 2024-12-05

**Authors:** Flavia Cristina Al-Akel, Lacramioara Eliza Chiperi, Vas Krisztina Eszter, Anca Bacârea

**Affiliations:** 1 Physiopathology Department, George Emil Palade University of Medicine, Pharmacy, Sciences and Technology, 540142 Targu Mures, Romania; alakelcristina@gmail.com (F.C.A.-A.); anca.bacarea@umfst.ro (A.B.); 2 Department of Pediatric Cardiology, Emergency Institute for Cardiovascular Diseases and Heart Transplant, 540136 Targu Mures, Romania; 3 Pediatrics Department, George Emil Palade University of Medicine, Pharmacy, Sciences and Technology, 540142 Targu Mures, Romania; 4 Department of Laboratory Medicine, County Hospital, 530173 Miercurea Ciuc, Romania; krisztinaesztervas@gmail.com; 5 Department of Laboratory Medicine, Emergency County Hospital, 540136 Targu Mures, Romania

**Keywords:** *Streptococcus salivarius*, probiotic, children, *Streptococcus salivarius* M18, *Streptococcus salivarius* K-12, *Streptococcus salivarius* 24SMB

## Abstract

Background: This systematic review aimed to synthesize the existing evidence on the use of *Streptococcus salivarius* (*S. salivarius*) probiotics as prophylactic or therapeutic tools for pediatric oral, dental, and respiratory diseases. Methods: A comprehensive search was carried out across multiple databases using the following terms: *S. salivarius*, probiotic, children, pediatric. Results: The systematic literature search identified 613 publications, which were meticulously screened, and, ultimately, 15 suitable citations were included in this systematic review. Three strains of *S. salivarius* (M18, K-12, 24SMB) were used, and they all demonstrated positive benefits in pediatric pathology. Conclusions: Administration of *S. salivarius* has benefits, is effective, and is convenient (cost-effective) in pediatric prophylaxis. Oral administration as a chewable tablet or powder of *S. salivarius* M18 for 3 months is able to reduce the incidence of black stains, plaque, and tooth decay in children. *S. salivarius* K-12 treatment decreased the occurrence of pharyngeal, recurrent, and streptococcal disease, and the benefits also extend to a reduction of nonstreptococcal diseases, including tracheitis, viral pharyngitis, rhinitis, flu, laryngitis, acute otitis media, and enteritis. Administration of *S. salivarius* 24SMB as an intranasal spray was able to reduce the risk of acute otitis media in children prone to this condition.

## 1. Introduction

The term “*Streptococcus*” (S) is attributed to the Austrian surgeon Theodor Billroth [1], who, in 1874, published a paper about these microorganisms, describing them as small organisms found isolated, in pairs, or in chains. Conditions such as pharyngitis, erysipelas, scarlet fever, puerperal fever, or wound infections troubled many doctors throughout history, some of them being described even by Hippocrates in 400 years BC. These infections are still present today in human pathology, especially in children.

Genus *Streptococcus* includes 126 species, as established by the International Committee on Systematics of Prokaryotes, one of them being represented by *S. salivarius*, which also includes two subspecies: *S. salivarius* subsp. *salivarius* (Andrewes and Horder 1906), Farrow and Collins 1984, and *S. salivarius* subsp. *thermophilus* (Orla-Jensen 1919), Farrow and Collins 1984 [2,3].

*Streptococcus salivarius* (*S. salivarius*), a Gram-positive bacteria, is one of the first bacteria that colonizes the human oral cavity and the upper respiratory tract right after birth [4]. *S. salivarius* is a commensal bacteria with an important probiotic role. Probiotics are *live bacteria and yeasts that have beneficial effects on the human body. S. salivarius* produces bacteriocins, which are antimicrobial peptides that can inhibit the growth of *S. pyogenes*, a key pathogen in pharyngeal, ear, and dental infections [5].

There are different strains of *S. salivarius* with probiotic roles. They differ in genomic DNA and the type of bacteriocins they produce. *S. salivarius* K-12 and M18 are two of the most widely studied probiotic strains, and each has unique antimicrobial properties. *S. salivarius* K-12 produces bacteriocins salivaricins A2 and B. It secretes bacteriocins with antagonistic inhibitory activity against *Streptococcus pyogenes* and partially against *Moraxella catarrhalis*, *Haemophilus influenzae*, and *Streptococcus pneumoniae* [6]. *S. salivarius* M18 secretes bacteriocins A2, 9, MPS, and M, and through these bacteriocins, it offers beneficial modulatory effects within the oral microbiome, inhibiting pathogenic bacteria such as *Streptococcus pyogenes* [7]. *S. salivarius* 24SMB is characterized by blpU bacteriocin production with antimicrobial activity against pathogens like *Streptococcus pyogenes* and *Streptococcus pneumoniae* [8].

Both *S. salivarius* K-12 and *S. salivarius* M18 are safe, non-pathogenic commensals from the human oral cavity, an important characteristic for pediatric use. Their safety profile was validated in early-stage research [9]. *S. salivarius* K-12 was selected for its ability to produce bacteriocin-like inhibitory substances (BLISs), which actively inhibit *Streptococcus pyogenes* (a key agent of streptococcal pharyngitis). This is a significant part of its mode of action. *S. salivarius* K-12 and *S. salivarius* M18 have the ability to colonize the upper respiratory tract and outcompete pathogens through niche competition and immune modulation [10].

This systematic review critically assesses the literature, identifies existing knowledge, and aims to provide an overview of the current evidence on the use of *S. salivarius* probiotics as a tool for the prevention of oral, dental, and respiratory diseases in pediatrics.

## 2. Materials and Methods

Using the standard methodology outlined by the Cochrane Collaboration, a comprehensive search was carried out across multiple databases, such as PubMed, Medline, Science Direct, and Google Scholar, covering their entire histories up to August 2024. The search was conducted using the following terms: *S. salivarius*, probiotic, children, and pediatric. Three strains usually used in prophylaxis were found in available studies, so the research was extended to include the following terms: *S. salivarius* M18, *S. salivarius* K-12, *S. salivarius* 24SMB. The results were recorded according to the guidelines specified by the Preferred Reporting Items for Systematic Reviews and Meta-Analyses (PRISMA) in 2020 [11].

We searched through theses, books, clinical studies, clinical trials, meta-analyses, randomized controlled trials, reviews, and systematic reviews. We included retrospective and prospective studies that evaluated the role of *S. salivarius* in children’s health, including dental health prevention of respiratory tract infections, otitis, and other diseases. Our review included studies involving populations of neonates, infants, children, and adolescents. We excluded studies that involved food enriched with probiotics, combined strains of probiotics, in vitro studies, and animal studies. A lot of articles were found that evaluated the role of *S. salivarius* as a probiotic in combination with other bacteria or a combination of more than one strain of *S. salivarius*, but those studies were excluded. Also, we excluded studies with inadequate data, population, or study protocols.

We recorded patient characteristics such as age, comorbidities, the intervention that the patients undertook, the comparator, and the setting in which the child was assessed.

Two reviewers conducted the research, achieving more than 95% inter-rater agreement. Only the articles the reviewers agreed on were included.

We used Microsoft Office version 2019 to synthesize and organize the data. The results are presented as intervals or means with standard deviations based on the data provided in each article.

## 3. Results

**Figure 1:**
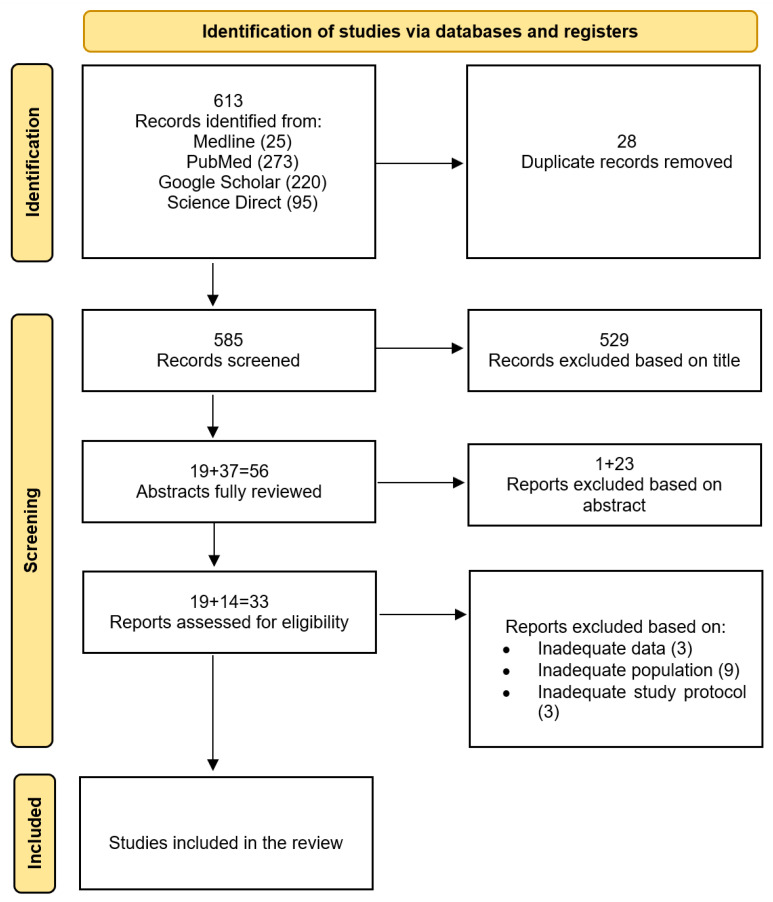
PRISMA 2020 flow diagram for new systematic review: study selection process.

The systematic literature search identified 613 publications, which were meticulously screened based on their titles and abstracts. Twenty-eight duplicates were excluded using Mendeley. After excluding non-relevant studies and duplicates, 33 articles were considered relevant and evaluated. Ultimately, 15 suitable citations were included in this systematic review: 3 articles concerning *S. salivarius* M18, 11 articles concerning *S. salivarius* K-12, and one article about *S. salivarius* 24SMB. Details of the selection process are presented in Figure 1.

The bias risk assessment of the included studies revealed some potential limitations represented by the fact that not all of the included studies were randomized trials; some were uncontrolled, prospective, or retrospective studies, as shown in Table 1, Table 2 and Table 3. However, the overall risk of bias was low due to the fact that 9 out of 15 included studies were randomized controlled trials. No important missing results were found in the included studies, so the confidence in the body of evidence was high.

The included studies evaluated a total of 2355 children with an age range between 6 months and 17 years old.

A sensitivity analysis was performed to evaluate the robustness of the synthesized results from the included studies. The synthesized results indicated similar effect sizes across age subgroups, suggesting that all groups may be affected by the oral *S. salivarius* administration intervention.

### 3.1. S. salivarius M18

For *S. salivarius* M18, three studies were found that evaluated its role in pediatric prophylaxis. All were randomized controlled studies. Table 1 summarizes key study characteristics, including patient populations, study designs, intervention details, and measured outcomes.

The studies included a total of 217 pediatric patients. Children included were preschoolers and school children and were evaluated as outpatients in dental clinics. The intervention was represented by the administration of a tablet containing *S. salivarius* M18 compared to no treatment or placebo. The studies concentrated on oral and dental health and demonstrated that oral administration of *S. salivarius* M18 for 3 months was able to reduce the incidence of black stains, plaque, and tooth decay in children.

**Table 1:** Characteristics of included studies evaluating *S. salivarius* stain M18 in pediatric prophylaxis.

Author, Publication Year	Study Design	Setting	Number of Children Included	Age	Intervention	Duration of Probiotic Administration	Comparator	Follow-Up Outcomes	Study Conclusions
Bardellini [12], 2020	Randomized controlled study	Dental clinic, Brescia, Italy	58 (29 intervention group, 29 control group)	4–10 years	A tablet containing 1 billion CFU of *S. salivarius* M18 once a day	3 months	No treatment	Black stains	Black stain formation in children could be prevented by administering * S. salivarius * M18
Di Pierro [13], 2015	Randomized, controlled study	Clinical practice, Milan, Italy	76 (38 treated group, 38 control group)	6–17 years	A tablet containing 1 billion CFU of *S. salivarius* M18 once a day	3 months	No treatment	Dental caries	The use of * S. salivarius * M18 increases the chances of avoiding new dental caries development in children
Burton [14], 2013	Randomized, double-blind, placebo-controlled study	Dental clinic, Dunedin, New Zealand	83 (40 treated group, 43 control group)	5–10 years	A tablet containing 3.6 billion CFU of *S. salivarius* M18 once a day	3 months	Placebo	Plaque score, gingival, and soft-tissue health	The plaque scores were significantly lower for children in the * S. salivarius * M18-treated group

Abbreviations: CFU = colony-forming unit; *S. salivarius* M18 = *S. salivarius* strain M18.

### 3.2. S. salivarius K-12

For *S. salivarius* K-12, eleven studies were found that evaluated its role in pediatric prophylaxis. They were retrospective/prospective studies or randomized controlled trials. Table 2 summarizes key study characteristics, including patient populations, study designs, intervention details, and measured outcomes.

Studies included a total of 2038 pediatric patients, including infants, toddlers, preschoolers, and school children, who were evaluated as outpatients or participants in daycare centers or schools. Not all children were healthy before inclusion in the study. Some had pathologies like PFAPA (periodic fever, aphthous stomatitis, and adenitis), recurrent GABHS (group A beta-hemolytic *Streptococcus pyogenes*), pharyngo-tonsillar infections, recurrent oral streptococcal disorders, chronic adenoiditis, or secretory otitis media. The intervention was represented by the administration of a tablet containing *S. salivarius* K-12, once per day for 3 months in the majority of the studies or for 6 or 9 months in others, compared with no treatment or placebo. The studies concentrated primarily on respiratory infections like pharyngitis, tonsilitis, and otitis media.

*S. salivarius* K-12 had no effect on culture-positive sore throats when given at school during the school day. However, when it was administered in the evening, after oral hygiene, it reduced episodes of streptococcal pharyngeal infections and/or tonsillitis in healthy subjects and also in children with recurrent oral streptococcal pathology. It was observed that benefits also extend to nonstreptococcal diseases (tracheitis, viral pharyngitis, rhinitis, flu, laryngitis, acute otitis media, and enteritis), which were also reduced. The use of *S. salivarius* K-12 probiotic leads to reduced antibiotic consumption or number of days under antibiotic and/or antipyretic therapy and also days of absence from school or work for parents. No protection against scarlet fever was detected.

Prophylactic administration of *S. salivarius* K-12 to children was associated with a reduced incidence of acute otitis media in five studies.

The daily administration of *S. salivarius* K-12 as a probiotic decreased the frequency of exacerbations of chronic adenoiditis and its complications and reduced the requirement for medication therapy. *S. salivarius* K-12 administration could also promote protection from SARS-CoV-2 infection and/or disease and could be beneficial in decreasing febrile episodes related to PFAPA syndrome and its associated symptoms.

**Table 2:** Characteristics of included studies evaluating *S. salivarius* strain K-12 in pediatric prophylaxis.

Author, Publication Year	Study Design	Setting	Number of Children	Age	Intervention	Duration of Probiotic Administration	Comparator	Follow-Up Outcomes	Study Conclusions
Spagnolo [15], 2024	Retrospective study	Outpatients with history of PFAPA, Catanzaro, Greece	117 (no control grup)	6 months–9 years	A tablet containing 1 billion CFU of *S. salivarius* K-12 once a day	6 months	None	Febrile attacks	The use of *S. salivarius* K-12 could be beneficial in decreasing febrile episodes related to PFAPA syndrome and its associated symptoms
Di Pierro [16], 2021	Randomized, controlled trial	School children, Milan, Italy	128 (64 treated group, 64 control group)	7.7 ± 3.2 years	1 daily dose of a * S. salivarius * K-12 product	3 months	No treatment	SARS-CoV-2 infection	Oral administration of oral-colonizing bacteria could afford protection from SARS-CoV-2 infection and/or disease
Doyle [17], 2018	Quasi-randomized (based on odd or even birthdates) study	School children, New Zealand	801 (411 treated group, 390 control group)	5–14 years	A tablet containing 2.5 billion CFU of *S. salivarius* K-12 once a day	One school year	Placebo	Group A streptococcus pharyngitis	* S. salivarius * K-12 had modest, nonsignificant effects on culture-positive sore throats when given at school, during the school day
Di Pierro [18], 2018	Retrospective study	Children living in Genoa area, Italy	133 (133 treated group, same 133 as control group)	3–14 years	A tablet containing 1 billion CFU of *S. salivarius* K-12 once a day	October–December 2015, for 90 consecutive days, and then from April–June 2016 for another 90 days	No treatment	Streptococcal and viral pharyngo-tonsillitis and AOM	* S. salivarius * K-12, if administered for two trimesters out of 12 months, is associated with a reduced incidence of pharyngitis and AOM in pediatric subjects with non-recurrent streptococcal infection
Di Pierro [19], 2016	Multicentre, open-label, randomized, controlled clinical trial	Children from area of Milan, Italy	222 (111 treated group, 111 control group)	33–45 months	A tablet containing 1 billion CFU of *S. salivarius* K-12 once a day	3 months	No treatment	Streptococcal disease (pharyngeal infection, scarlet fever) and AOM	The daily administration of K-12 to children attending their first year of kindergarten was associated with a significant reduction in episodes of streptococcal pharyngitis and AOM; no protection against scarlet fever was detected
Gregori [20], 2016	Retrospective observational analysis	Children with recurrent GABHS pharyngo-tonsillar infections from Piacenza, Italy	130 (76 treated group, 54 control group)	3–7 years	A tablet containing 1 billion CFU of *S. salivarius* K-12 once a day	3 months	No treatment	GABHS pharyngo-tonsillar infections	Observations are supportive of the use of probiotic * S. salivarius * K-12 for the control of recurrent GABHS pharyngo-tonsillar infections in children; the use of this probiotic could lead to reduced antibiotic consumption
Di Pierro [21], 2016	Multicenter, open, non-randomized, controlled clinical trial	Children from area of Milan, Italy	124 (48 treated group, 76 control group)	3–10 years	A tablet containing 1 billion CFU of *S. salivarius* K-12 once a day	3 months	No treatment	Episodes of tracheitis, viral pharyngitis, rhinitis, flu, laryngitis, AOM, enteritis, and stomatitis	Reduction in the occurrence of pharyngeal, recurrent streptococcal disease was observed; the benefits may also extend to a reduction of nonstreptococcal diseases, including tracheitis, viral pharyngitis, rhinitis, flu, laryngitis, AOM, and enteritis
Karpova [22], 2015	Open randomized comparative study	Outpatients, children with chronic adenoiditis, Russia	219 (113 treated group, 106 control group)	6–7 years	* S. salivarius * K-12- based probiotic in combination with nasal douche	30 days	Nasal douche alone	Exacerbation of adenoiditis	Use of * S. salivarius * K-12 -based probiotic permits a decreased frequency of exacerbations of chronic adenoiditis and its complications in children and reduces the requirement for medication therapy
Di Pierro [23], 2015	Pilot, uncontrolled study	Children with secretory otitis media, outpatients, Milan, Italy	22 (no control group)	3—9 years	A tablet containing 1 billion CFU of *S. salivarius* K-12 once a day	3 months	None	AOM episodes	The results indicate a substantial reduction of AOM episodes and a positive outcome from the treatment for all of the clinical outcomes tested
Di Pierro [24], 2014	Multicenter, open, non-randomized, controlled clinical trial	Children with a diagnosis of recurrent oral streptococcal disorders, Milan, Italy	60 (30 treated group, 30 control group)	3–13 years of age	A tablet containing 1 billion CFU of *S. salivarius* K-12 once a day	3 months	No treatment	Streptococcal and viral pharyngitis and/or tonsillitis	Prophylactic administration of * S. salivarius * K-12 in children with a history of recurrent oral streptococcal disease resulted in a considerable reduction of episodes of both streptococcal and viral infections and reduced the number of days under antibiotic and/or antipyretic therapy and days of absence from school or work
Di Pierro [25], 2012	Prospective, comparative study	Children from daycare center, Italy	82 (45 treated group, 37 control group)	3–12 years	A tablet containing 1 billion CFU of *S. salivarius* K-12 once a day	3 months	No treatment	Streptococcal pharyngitis and/or tonsillitis and episodes of AOM	Prophylactic administration of * S. salivarius * K-12 in children with a history of recurrent oral streptococcal pathology reduced the number of episodes of streptococcal pharyngeal infections and/or tonsillitis as well as episodes of AOM

Abbreviations: AOM = acute otitis media; CFU = colony-forming unit; GABHS = group A beta-hemolytic streptococci; PFAPA = periodic fever, aphthous stomatitis, pharyngitis, and cervical adenitis syndrome.

### 3.3. S. salivarius 24SMB

For *S. salivarius* 24SMB, only one study was found that evaluated its role in pediatric prophylaxis. Some other studies included a probiotic combination of *S. salivarius* 24SMB and other strains, but those studies were excluded. Table 3 summarizes key study characteristics, including patient populations, study designs, intervention details, and measured outcomes.

The children included were preschoolers with a history of recurrent acute otitis media (at least three episodes in the preceding 6 months or at least four episodes in the preceding 12 months) who were evaluated as outpatients in the pediatric department. The intervention was represented by the administration of intranasal suspension of *S. salivarius* 24SMB as spray twice daily for 5 days each month (3 consecutive months) after an initial course of antibiotics (amoxicillin–clavulanate) in order to reduce competing nasopharyngeal flora. It was compared with a placebo nasal spray. The study demonstrated that intranasal administration of *S. salivarius* 24SMB as a spray was able to reduce the risk of acute otitis media in otitis-prone children.

**Table 3:** Characteristics of included study evaluating *S. salivarius* stain 24SMB in pediatric prophylaxis.

Author, Publication Year	Study Design	Setting	Number of Children Included	Age	Intervention	Duration of Probiotic Administration	Comparator	Follow-Up Outcomes	Study Conclusions
Marchisio [26], 2015	Prospective, randomized, double-blind, placebo-controlled study	Outpatients, Milan, Italy	100 children with history of recurrent AOM (50 treated, 50 placebo)	1–5 years	Intranasal suspension of 100 billion CFU of * S. salivarius * 24SMB twice daily for 5 days each month	3 consecutive months	Placebo	Followed monthly for 6 months for episodes of AOM	This study revealed the ability of intranasally administered * S. salivarius * 24SMB to reduce the risk of AOM in otitis-prone children

Abbreviations: AOM = acute otitis media; CFU = colony-forming unit; *S. salivarius* 24SMB = *S. salivarius* 24SMB.

## 4. Discussion

The current systematic review synthesizes the existing evidence on the use of *S. salivarius* probiotics as a prophylactic tool for pediatric otitis media, respiratory and other infections, and dental health. We provide an overview of the current evidence regarding probiotic effects in preventing acute otitis media (AOM), pharyngitis/tonsilitis/adenoiditis, SARS-CoV2 and other infections, periodic fever, aphthous stomatitis, pharyngitis and adenitis (PFAPA) syndrome attacks, and oral and dental problems.

*S. salivarius* K-12 is suggested to exert its effects by eliciting no proinflammatory response, stimulating an anti-inflammatory response, modulating genes involved in adhesion to the epithelial layer, and maintaining homeostasis. In this way, *S. salivarius* K-12 may ensure its tolerance by the host, persistence on the epithelial surface, and active protection against inflammation and apoptosis induced by pathogens [27].

*S. salivarius* M18 can modulate inflammatory factors produced by human gingival fibroblasts exposed to common dental pathogens, as well as in healthy volunteers, and leads to changes in the salivary microbiome and alterations in secreted cytokines [28].

*S. salivarius* 24SMB is capable of inhibiting the biofilm formation of selected pathogens and even dispersing their pre-formed biofilms in the upper respiratory tract. Diffusible molecules secreted by it, along with the lowered pH of the medium, were found to be involved in the mechanisms underlying the anti-biofilm activity [29].

### 4.1. Acute Otitis Media

AOM is a burdensome disease in pediatric pathology. Studies have described that 80% of children present at least one episode of AOM during their childhood [30], and 80–90% of preschoolers have already had at least one episode of secretory otitis media when they start school [31]. Regarding recurrence, studies described that in 50% of patients, a new episode of AOM appeared in the following 3 months after the last episode of AOM [32].

Lactobacilli can be, naturally, minimally found in nasopharyngeal microbiota and extensively found in gastrointestinal microbiota, and large studies were performed regarding the use of lactobacilli as prophylaxis of AOM and concluded that it is ineffective [33].

*S. salivarius* K-12 produces salivaricins A2 and B, which have been shown in vitro to inhibit the growth of *S. pyogenes* or *S. pneumoniae*, *Moraxella catarrhalis*, and *Haemophylus influenzae*, the etiological agents of AOM and pharyngo-tonsillar infections [6,34]. Other studies have already shown that after oral administration of *S. salivarius* K-12, it colonizes the upper respiratory tract, mouth, nasopharynx, and adenoids and is able to persist locally up to 1 month after the last dose was ingested [35,36]. *S. salivarius* K-12, also named *S. salivarius*, produces anti-*Streptococcus pyogenes* bacteriocin-like inhibitory substances (BLIS) and has been isolated from the oral cavity of a healthy kid in New Zealand [37].

The alfa-hemolytic strain Ss 24SMB also produces bacteriocin-like substances and has been isolated from the nasopharyngeal swab of a healthy child [38].

Di Pierro et al. [18] conducted a study in 133 children with non-recurrent ear pathology, studying the potential benefits of oral administration of *S. salivarius* K-12. Inclusion criteria were represented by children 3 to 14 years old who have had at least one episode of AOM. They retrospectively analyzed the number of episodes of AOM and then compared this with the incidence of AOM in the same target group after administration of *S. salivarius* K-12 in this fashion: from October to December 2015 (for 90 consecutive days) and from April to June 2016 (for another 90 days), after teeth brushing, one tablet of *S. salivarius* K-12 was dissolved slowly in the mouth, before night sleep and no liquids or food consumed afterward. The results of the study showed a 70% reduction in the number of episodes of AOM after *S. salivarius* K-12 administration compared with the period with no treatment in the same target group. The study also showed that on *S. salivarius* K-12 prophylaxis, there was reduced use of antibiotics, antipyretics, and anti-inflammatory drugs by more than 80% and a decreased number of absences from school by 85% or work for parents by 75%. Another very important aspect demonstrated was that *S. salivarius* K-12 is a safe probiotic with excellent compliance and tolerability, with only 1 out of 133 children studied experiencing one episode of mild bronchospasm.

Di Pierro et al. [25] conducted another study on 82 children aged 3–12 years, treated for a period of 90 days with one slow-release tablet per day, taken orally, containing 5 billion colony-forming units of *S. salivarius* K-12. After 3 months of *S. salivarius* K-12 administration, another 6 months of follow-up were used in order to observe if the protective role of *S. salivarius* K-12 persists. The number of episodes of AOM was reduced by 40% compared to the previous year’s infection rates, and the incidence of ear infections during the follow-up period was reduced by 65% compared with controls. No side effects and good tolerability were reported. The conclusion of the study was that it is feasible to administer this harmless strain of probiotics as a prophylaxis for AOM.

Another study (multicentre, open-label, randomized, controlled clinical trial) conducted by Di Pierro et al. [19] aimed to describe what happened with 3-year-old children in their first year of kindergarten. They randomly divided 222 children, aged 33–45 months, into a treated group and a control group, numerically equal. The target group received daily treatment with 1 billion CFUs of *S. salivarius* K-12 after brushing their teeth, just before night sleep, with no food or drink after the administration of the tablet, for half a year. During the 6 months, AOM incidence was 44.1% in the treated group and 80.2% in the control group. After this treatment period, another 3 months of follow-up were used to observe the pattern of disease incidence. New onset of AOM was 13.8% in the treated group, compared to 41.3% in the controls. Treatment tolerance was carefully observed during this entire period (6 months of treatment and 3 months of follow-up), and no apparent side effects were noticed. The conclusion of this study was that daily administration of *S. salivarius* K-12 for 6 months in their first year of preschool enrolment is beneficial, with a significant reduction in number of episodes of AOM.

A pilot, uncontrolled study conducted by the same Di Pierro et al. [23] aimed to study secretory otitis media (SOM) among children. SOM is considered a sequel of AOM, characterized by middle ear fluid that persists for more than 3 months. Twenty-two children (3–9 years old) with a recent history of AOM and SOM were treated daily for 3 months with 1 billion CFUs of *S. salivarius* K-12. After treatment, subjects were evaluated by tympanometry, otoscopy, tone audiometry, tonsillar exam, and nasal endoscopy for the evaluation of SOM progression. The study pointed out a significant reduction in AOM episodes, concluding that *S. salivarius* K-12 may also play a role in SOM occurrence and severity of episodes in children. AOM incidence was reduced by 40% during the 90 days of treatment compared with the previous year, audiometry improved by 50–60%, and bilateral otoscopy improved by 40%. Tympanometry also improved. Adenoid vegetations obstructing the eustachian tube decreased by 30%, and the size of the tonsils was improved by 40%. The researchers carefully observed the compliance and tolerability for treatment and potential side effects, concluding a good safety profile for *S. salivarius* K-12.

Marchisio et al. [26] studied another strain of *S. salivarius*, *S. salivarius* 24SMB, an alpha-hemolytic strain, trying to prove its role in otitis prophylaxis for AOM-prone children. They conducted a prospective, double-blind, placebo-controlled trial involving 100 subjects aged between 1 and 5 years, with a personal history of otitis media recurrency, defined as at least three episodes of AOM in the last 6 months or four or more episodes in the last year, with the last episode 2 to 8 weeks before study inclusion. Before the study, all children were treated with amoxicillin + clavulanic acid for 10 days. Subjects were equally and randomly divided 1:1, either into study or control group, receiving *S. salivarius* 24SMB intranasally or placebo, twice per day, 5 days consecutively in a month, for a period of 3 consecutive months. Researchers considered a 25-day spacing period necessary between each monthly administration (5 consecutive days of treatment or placebo followed by 25 days of pause). The posology administered was 5 × 10^9^ CFU of *S. salivarius* 24SMB as a nasal spray in each nostril, two times per day. Placebo spray contained saline solution with the same flavor and color as the *S. salivarius* 24SMB spray. All subjects were followed once per month to monitor the occurrence of AOM, for half a year. AOM diagnosis was based on a clinical otorhinolaryngology exam, followed by pneumatic otoscopy and tympanometry in the case of uncertain diagnosis. Results of the study showed that the number of subjects without new episodes of AOM was higher (30%) in the study group compared with the control group (14.9%). Of the children who received *S. salivarius* 24SMB intranasally, 70% needed antibiotic treatment during the study period, compared with 83% of the subjects in the placebo group. Safety and tolerability of the product were studied in a trial on an adult healthy population, and no adverse effects were reported [39]. According to Marchisio et al. [26], the product was safe and easy to tolerate, but 42% of children in the study group reported adverse events, only locally, at the site of administration, and not severe (for example, local burning, sneezing, itching, cough), compared with 14.9% in the placebo group. All of these events disappeared 24 h after the last dose. They concluded that *S. salivarius* 24SMB administered intranasally has a good outcome, being able to reduce the risk of new episodes of AOM in otitis-prone subjects. This method of prevention, using *S. salivarius* 24SMB probiotic, has already shown a better outcome than other prophylactic methods used previously: vaccines (pneumococcal, influenza) or vitamin D [40,41].

### 4.2. Pharyngitis, Tonsillitis, Adenoiditis

Another important category of frequent infections in pediatric pathology is respiratory infections like pharyngitis, tonsilitis, or adenoiditis. *S. salivarius* prophylaxis also has an important role in this domain.

Doyle et al. [17] studied the prophylaxis of Group A β−hemolytic *Streptococcus* (GABHS) pharyngitis in children aged 5 to 14 years old at risk of rheumatic fever using *S. salivarius* K-12 as an oral probiotic. They published a quasi-randomized study on 1314 subjects, divided into either a study group or a control group. Subjects in the study group received 2.5 × 10^9^ CFU *S. salivarius* K-12 per lozenge, and the control group received a daily placebo during school time from Monday to Friday for a period of one school year. Sixty-one percent of subjects experienced a sore throat at least once, so pharyngeal swabs were taken. Of the total number of swabs, 52.1% were taken from children in the study group, and 7.8% were positive versus 47.8% taken from subjects in the placebo group, from which 8.8% were positive for group A streptococcus. The results of the study showed no statistically significant reduction in the number of positive swabs for the study group receiving *S. salivarius* K-12 compared with the control group. They concluded that the administration of oral *S. salivarius* K-12 probiotics during school days, at school time, is of no benefit for group A streptococcus pharyngitis prophylaxis.

Gregori et al. [20] conducted an observational, retrospective study on the use of *S. salivarius* K-12 oral as prophylaxis for recurrent GABHS pharyngo-tonsillitis. This study involved children with a personal history of recurrent GABHS pharyngo-tonsillitis in the last 6–12 months prior to inclusion in the study. Children included in the study group were treated with *S. salivarius* K-12 once daily for 90 days. All subjects were monitored for one year after inclusion in the study. Results have shown that children treated with *S. salivarius* K-12 had significantly lower rates of GABHS infection compared with the control group both in the 90-day period of trial and in the following 9 months of monitoring. They concluded that *S. salivarius* K-12 is a reliable method of prophylaxis for recurrent GABHS pharyngo-tonsillitis and that using this probiotic can decrease antibiotic consumption for this particular group of patients.

Another study already mentioned in this review in the AOM section [25] provided the importance of *S. salivarius* K-12 use as a prophylaxis method in preventing recurrent *Streptococcus pyogenes* pharyngo-tonsillitis in the pediatric population. Patients were treated with *S. salivarius* K2 in the form of one slow-release tablet taken orally, containing 5 billion CFU of *S. salivarius* K-12 per tablet daily for 90 days. The results of the study showed a 90% reduction in the number of streptococcal pharyngitis episodes for children who completed the treatment with *S. salivarius* K-12, compared with pharyngeal streptococcal infection rates in the previous year, prior to study enrolment. In the 6-month follow-up period after the study, the reported incidence of pharyngitis was reduced by 65% for the subjects who completed the 90-day trial of product administration. They concluded that using *S. salivarius* K-12 as a prophylaxis for oral streptococcal pharyngeal disease has a very good outcome in children with a history of recurrent disease, reducing the number of episodes of pharyngitis and/or tonsillar infection during and 6 months after treatment.

A study previously mentioned in the AOM section, conducted by Di Pierro et al. [19], studied the possible effect on the incidence of scarlet fever and streptococcal pharyngitis/tonsillitis in three-year-old kindergarten subjects after the administration of *S. salivarius* K-12. Children with non-recurrent disease, attending their first year of kindergarten were included in the study, and the results during the trial period showed that subjects in the treated group, receiving BLIS K-12 daily for a period of 6 months, had an incidence of scarlet fever of 9% and an incidence for streptococcal pharyngo-tonsillitis of 16%, compared with the control group, where the incidences were 4% for scarlet fever and 48% for pharyngo-tonsillitis. Also, subjects in each group were followed-up for a period of 3 months, and the incidences of disease in the treated group were 15% for streptococcal pharyngo-tonsillitis and 0% for scarlet fever, versus the control group of 26% for streptococcal pharyngo-tonsillitis and 6% for scarlet fever. They concluded that *S. salivarius* K-12 administered daily for 6 months is associated with a significant reduction in streptococcal pharyngo-tonsillitis incidence but has no effect on scarlet fever prophylaxis.

Di Pierro et al. [24] published a paper describing the results of the prophylactic use of *S. salivarius* K-12 in pharyngo-tonsillitis of viral or streptococcal etiology. The study enrolled 61 subjects aged 3 to 13 years diagnosed with oral recurrent streptococcal disease. In the study group, children were treated with one *S. salivarius* K-12 slow-release tablet per day containing 1 billion CFU of *S. salivarius* K-12, taken orally daily for a period of 90 days. The results of the study showed a 96% reduction in the number of episodes of streptococcal pharyngitis for children in the study group compared with the previous 12 months before treatment. The control group exhibited no difference regarding this aspect (only 7% reduction in streptococcal infection rates). The study group presented an 80% decrease in the incidence of viral oral infections compared with the previous year before treatment. No significant difference was noticed in the control group (14%) compared with the previous year. Another observation of the study was that only 30 days of antibiotic treatment were reported in the study group, compared with 900 days of antibiotic treatment for the controls. Also, for the children in the study group, 16 days of antipyretic therapy were reported compared with 228 days reported for the control group. Regarding days missing from school or work (for parents), the study reported only 16 days for the treated group compared with 228 days for the control group subjects and their parents. There were no side effects reported, and the product had good tolerability. Only one subject reported bad palatability of the *S. salivarius* K-12 tablet and dropped out of the study. The conclusion of the study was that the use of *S. salivarius* K-12 product as a prophylaxis in children with oral streptococcal recurrent disease was associated with considerably lower rates of viral and streptococcal infections, reduced number of missing school days for patients or work days for parents, and considerably reduced antibiotic and/or antipyretic need in terms of days.

Another study, conducted by Di Pierro al. [18], previously mentioned in the acute otitis media section above, analyzed the effect of *S. salivarius* K-12 on streptococcal and viral pharyngo-tonsillitis incidence for non-recurrent streptococcal disease in children when administered in 2 trimesters out of 1 year. Results showed that *S. salivarius* K-12 use reduced pharyngo-tonsillitis incidence by 90%.

Regarding chronic adenoiditis in children, Karpova et al. [22] conducted an open, comparative, randomized study with the purpose of improving prophylaxis methods for this particular disease. They included 219 subjects, aged 6 to 7 years old, with chronic adenoiditis. The study group was treated with a *S. salivarius* K-12 product associated with nasal douche for a period of 30 days, and the control group was treated only with nasal douche. The results showed that after the last dose of treatment, the exacerbation episodes of adenoiditis occurred in 49.6% of the subjects in the treated group compared with 88.7% of children in the control group. Also, acute sinusitis was diagnosed after 3 months of follow-up in 3.5% of subjects in the study group versus 13.2% in the control group. The conclusion of this study was that using an *S. salivarius* K-12-based probiotic is associated with lower rates of exacerbations of adenoiditis in children with chronic disease and is able to decrease the number of acute sinusitis episodes in children with chronic adenoiditis.

### 4.3. PFAPA, SARS-CoV-2, and Other Infections

PFAPA syndrome is the most frequent cause of fever, with a periodic pattern in childhood, and usually affects children younger than 5 years who have an autolimitated evolution 3 to 5 years after the onset [42]. Studies have shown that *S. salivarius* K-12 is able to antagonize PFAPA syndrome attacks by elevating salivary gamma-interferon concentrations without modifications in TNF-alpha or IL-1 beta levels and by lowering IL-8 release [6]. Spagnolo et al. [15] published a paper after studying the effects of *S. salivarius* K-12 administration in a cohort of subjects with PFAPA syndrome. The aim of the study was to observe the effects of *S. salivarius* K-12 administration on febrile attacks; 117 PFAPA children aged between 6 months and 9 years old were enrolled. Before *S. salivarius* K-12 treatment, the febrile attacks appeared recurrently every 26.1 ± 11.5 days, and the duration of episodes was 4.1 ± 1.4 days. The temperature during the attacks was 39.8 ± 0.7 °C. After administering *S. salivarius* K-12 for a period of 6 months, the febrile attacks appeared every 70 ± 53.1 days; there was a reduction in the duration of the episode at 3.3 ± 1.6 days, and the highest temperature during fever episodes was lower than before, at 39.1 ± 1.1 °C. Regarding febrile attack-associated symptoms, they reported a reduction in the frequency of exudative pharingo-tonsillitis from 88% to 61.5% after *S. salivarius* K-12 treatment, a reduction in aphthous stomatitis from 68.4% to 40.2% after *S. salivarius* K-12 administration and a reduction in cervical lateral lymphadenopathy from 71% to 38.5% after *S. salivarius* K-12 treatment. They concluded that the administration of *S. salivarius* K-12 is effective in reducing the number and duration of febrile attacks and incidence of other characteristic symptoms related to PFAPA syndrome in pediatric patients, potentially with positive outcomes on their quality of life and further need for other medications.

Di Pierro et al. [16] published a paper describing the effects of *S. salivarius* K-12 administration in reducing SARS-CoV-2 infection rates in children. During the study, they observed that only in the control group not treated with *S. salivarius* K-12 children had nasal swabs positive for SARS-CoV-2 infection, agreeing with results proposed by other authors on the use of oral probiotic *S. salivarius* K-12 as a prophylaxis in reducing SARS-CoV-2 episodes [43].

Di Pierro et al. [21] published a paper focusing on *S. salivarius* K-12’s ability to protect against nonstreptococcal infections. The study group received *S. salivarius* K-12 daily for a period of 90 days, and the control group received a placebo. Both of the groups were monitored in terms of episodes of infections during the 90 days of treatment and 9 months after completing the treatment. Results showed a statistically significant reduction in episodes of nonstreptococcal disease like tracheitis, flu, rhinitis, pharyngitis of viral etiology, AOM, laryngitis, and enteritis.

### 4.4. Oral and Dental Health

When describing prophylaxis in dental health, the vast majority of studies use *S. salivarius* M18 [12,13,14] in the same posology: a Tablet of *S. salivarius* M18 daily for a period of 3 months. Bardellini et al. [12] reported that black stain formation could be prevented in children aged 4 to 10 years old by *S. salivarius* M18 prophylactic administration. Di Pierro et al. [13] reported that *S. salivarius* M18 is a good prophylactic method against the development of new dental caries in children aged 6 to 17 years old. Burton et al. [14] studied the use of *S. salivarius* M18 on children aged 5 to 10 years old and reported significantly reduced plaque scores in subjects treated with *S. salivarius* M18, compared with subjects who received placebo.

### 4.5. Limitations

The limitations of this systematic review arise from the constraints present in the included studies. Not all of the included studies were randomized, placebo-controlled, and blinded. In some studies, even a control group was missing. Furthermore, some studies included a relatively small number of children. The conclusions of this systematic review rely significantly on the quality of the included studies. If studies exhibit methodological flaws, such as small sample sizes, insufficient controls, or inadequate protocol, the reliability of the review’s findings could be jeopardized.

A source of bias could be represented by one of the inclusion criteria, which was English language restriction. Another source of bias is represented by the variability in study designs, populations, interventions, and outcomes.

Regarding future research directions, we outline that randomized controlled trials with larger populations and diverse demographics would be important to validate the findings presented in this review.

The findings of this systematic review, despite its limitations, suggest that oral administration of *S. salivarius* could represent a valuable option for preventing pediatric diseases and emphasizes that the prophylactic use of *S. salivarius* could reduce healthcare costs, improve the quality of life for children and their families, and lower antibiotic use, a very important consideration in the context of rising antibiotic resistance.

## 5. Conclusions

Administration of *S. salivarius* is beneficial, effective, and convenient (cost-effective) in pediatric prophylaxis. Three strains of *S. salivarius* were used as a main method of treatment, and they all proved to be of help in pediatric pathology.

Oral administration of *S. salivarius* M18 for 3 months is able to reduce the incidence of black stains, plaque, and tooth decay in children.

Regarding pharyngitis and otitis, the majority of studies showed that *S. salivarius* K-12 treatment decreased the occurrence of pharyngeal, new or recurrent streptococcal disease, and the benefits also extend to a reduction of nonstreptococcal diseases, including tracheitis, viral pharyngitis, rhinitis, flu, laryngitis, acute otitis media, and enteritis. Some benefits were also observed in COVID-19 infection, and it also decreased the frequency of exacerbations of chronic adenoiditis. One study demonstrated that using *S. salivarius* K-12 as a prophylactic treatment can decrease the number of febrile episodes related to PFAPA syndrome and its associated symptoms.

The administration of *S. salivarius* 24SMB as an intranasal spray is able to reduce the risk of AOM in children prone to this condition.

## Data Availability

Data is found in the cited studies.
